# Organizing integrated care in a university hospital: application of a conceptual framework

**DOI:** 10.5334/ijic.1529

**Published:** 2014-06-19

**Authors:** Runo Axelsson, Susanna Bihari Axelsson, Jeppe Gustafsson, Janne Seemann

**Affiliations:** Department of Sociology and Social Work, Centre for Organization, Management & Administration, Aalborg University, Aalborg, Denmark; Department of Sociology and Social Work, Centre for Organization, Management & Administration, Aalborg University, Aalborg, Denmark; Department of Business and Management, Aalborg University, Aalborg, Denmark; Department of Sociology and Social Work, Centre for Organization, Management & Administration, Aalborg University, Aalborg, Denmark

**Keywords:** process orientation, integrated care, organizational models, patient flows

## Abstract

**Background and aim:**

As a result of New Public Management, a number of industrial models of quality management have been implemented in health care, mainly in hospitals. At the same time, the concept of integrated care has been developed within other parts of the health sector. The aim of the article is to discuss the relevance of integrated care for hospitals.

**Theory and methods:**

The discussion is based on application of a conceptual framework outlining a number of organizational models of integrated care. These models are illustrated in a case study of a Danish university hospital implementing a new organization for improving the patient flows of the hospital. The study of the reorganization is based mainly on qualitative data from individual and focus group interviews.

**Results:**

The new organization of the university hospital can be regarded as a matrix structure combining a vertical integration of clinical departments with a horizontal integration of patient flows. This structure has elements of both interprofessional and interorganizational integration. A strong focus on teamwork, meetings and information exchange is combined with elements of case management and co-location.

**Conclusions:**

It seems that integrated care can be a relevant concept for a hospital. Although the organizational models may challenge established professional boundaries and financial control systems, this concept can be a more promising way to improve the quality of care than the industrial models that have been imported into health care. This application of the concept may also contribute to widen the field of integrated care.

## Background and aim

Process orientation has become something of a fashion in health care. It has been inspired by the ideas of New Public Management, which means an application of management methods from the private sector in the organizations of the public sector in order to improve their efficiency [1, 2]. In health care, as a result of New Public Management, artificial markets have been created through purchaser–provider split and commissioning of services [3]. In addition, many hospitals have applied concepts of process improvement from the private sector, for example, total quality management and business process reengineering [4, 5].

As part of this development, a number of industrial models of quality management have been translated to health care settings and used for process improvement, mainly in hospitals [6, 7]. Based on a systems approach, these models are concerned with measuring the time and costs of different processes in an organization in order to identify obstacles and reduce waste of resources. The most popular model at the moment seems to be Lean Production, which has been imported from the Japanese car manufacturing industry [8]. In Sweden, for example, 9 out of 10 hospitals claim that they have applied this model to some extent [9]. It is not clear, however, what results the application of this model has actually achieved. It is difficult to evaluate the effects of management models. Instead, there has been a discussion for many years about the merits and risks of management fashions in health care [10, 11].

At the same time as the application of industrial models in hospitals, there has been another development of process orientation in health care. This development has taken place within the health sector and it has been supported by the World Health Organization. Starting from the Ottawa Charter of Health Promotion [12] and the World Health Report [13], a concept of integrated care has been introduced in order to improve the quality and efficiency of health services. Integrated care has been defined as bringing together inputs, delivery, management and organization of services related to diagnosis, treatment, care, rehabilitation and health promotion in order to improve the access, quality, user satisfaction and efficiency of these services [14]. It has been described as a coherent set of methods and models on the funding, administrative, organizational, service delivery and clinical levels designed to create connectivity, alignment and collaboration within and between the cure and care sectors [15].

The development of integrated care started in the fields of health promotion and primary health care, but the concept has also been applied in other fields of health and social care like care of the elderly, care of chronic conditions and different forms of community care, including open psychiatric care and treatment of drug abuse. As a result, a number of process oriented models of integration have been developed for different diseases and patient or client groups, for example, shared care, integrated care pathways and chains of care [16–18]. Beside these more or less clinical models, there are also other professional, organizational and system models of integration [19]. In contrast to the industrial models of quality management, the models of integrated care are based mainly on experiences from the health sector of working across the boundaries of different medical specialties, different professions and different organizations dealing with the same group of patients or clients.

The industrial models and the models of integrated care have not only different origins, but they also represent different strategies of process orientation in health care. The industrial models are focusing on the measurement of time and costs of different health-related processes, while the models of integrated care are more concerned with the organization and management of patient care across professional and organizational boundaries. Moreover, the industrial models have mainly an intraorganizational perspective, concentrating on processes within an organization like for example a hospital, while the models of integrated care have an interorganizational as well as an intraorganizational perspective and sometimes even an intersectoral perspective, dealing with processes across many different organizations of health and welfare [20]. With this broad orientation, there has not been so much focus on hospitals – and they have been only partly involved – in the development of integrated care.

Against this background, the aim of the article is to discuss the relevance of integrated care for hospitals. The discussion is based upon a conceptual framework outlining a number of organizational models of integrated care on different levels of integration. It will be conducted against the background of preliminary data from a study of a Danish university hospital implementing a new organization for improving the patient flows of the hospital.

## Conceptual framework

According to the definitions of integrated care mentioned above, an important part of this concept concerns the management and organization of health services in order to create connectivity, alignment and collaboration within and between different services. The goal is to enhance quality of care and quality of life, consumer satisfaction and system efficiency for patients [15]. Integration is in this connection defined as bringing together different activities and organizational units for the benefit of the patients. This means organizing processes as well as structures. Moreover, integration can be horizontal as well as vertical. Horizontal integration means linking health services on the same organizational level, while vertical integration brings together services operating on different levels of the health system [14].

In the literature of institutional economics, integration has often been described in terms of markets and hierarchies [21]. Integration can be achieved either through the ‘invisible hand’ of market competition or through the more visible hand of a management hierarchy [22]. The management hierarchy means a top-down coordination of different actors, while competition on a market leads to contractual relations between purchasers and providers. According to the literature of organization and management, there is also a third way to achieve integration, namely through networks. This means a more or less voluntary cooperation or collaboration between different actors [23, 24]. Against this background, integrated care implies a matrix way of organizing, combining the vertical integration of a management hierarchy with the horizontal integration of a network. This means, for example, organizing teams or projects across existing professional or organizational boundaries [25, 26].

As mentioned before, there are a number of process-oriented models of integrated care. In addition to the clinical models of shared care, integrated care pathways and chains of care, there are also models which are more specifically oriented towards the organization and management of integrated care. These models can be roughly divided into interprofessional and interorganizational models of integration:

- The interprofessional models are dealing with integration of activities performed by different professionals or professional groups. These models include consultations and information exchange between professionals dealing with the same or similar patients [27, 28], more or less systematic meetings of different professionals involved in the treatment of a particular group of patients [29], and work in multidisciplinary teams of professionals with complementary skills and knowledge [30, 31].
- The interorganizational models are concerned with integration of different organizations or organizational units. Among these models are appointment of case managers for coordination of care on behalf of individual patients or patient groups [32], formal agreements on collaboration or partnerships between organizations involved in the same treatments [33, 34], co-location of professionals or organizational units dealing with the same patient groups [35] and financial coordination or pooling of resources from different organizations or units for the benefit of the patients [36, 37].

The distinction between these different types of organizational models is not so sharp, but the interorganizational models are focusing mainly on an ‘institutional’ level of integration, while the interprofessional models are more ‘person centered’ [38]. This means that they are operating on different levels of integration. Many of these models are also complementary, which means that they can be used in different combinations. For example, consultations and information exchange can be combined with most of the other models of integrated care. In the same way, case management is often combined with interprofessional meetings and/or multidisciplinary teamwork. Partnerships between organizations may also be combined with co-location as well as financial coordination [39].

Both the interprofessional and the interorganizational models may be more or less complex, depending on the number of organizational units and professionals involved. The complexity of the models may also be due to the ‘intensity’ in contacts, relationships and modes of work [40]. The interprofessional models may be more or less formalized in standard programmes, guidelines and protocols. In the same way, the interorganizational models may be more or less formalized in agreements, contracts and other structural arrangements. Although the models thus have different degrees of complexity and formalization, there is not one optimal model of integration, or one optimal combination of such models, that can be applied everywhere. However, one model or combination of models may be more appropriate than others in a certain context, depending most of all on the needs of the patients or clients served but also on the differentiation and fragmentation of the institutional environment [20].

## A university hospital in transition

### Context and methods

The Danish health care system is a public system, which is financed predominantly through general taxation. Primary health care is provided by approximately 3400 general practitioners and the 98 municipalities of the country. The general practitioners are family doctors who are paid on a combined capitation and fee-for-service basis. The municipalities are responsible for nursing homes and home care. They are also responsible for prevention, health promotion and rehabilitation. Secondary and tertiary care is provided by hospitals owned and run by the five regions of the country. There are four university hospitals in Denmark providing secondary and highly specialized tertiary care as well as medical education and research.

One of the university hospitals started in 2012 to reorganize its clinical structure in order to improve the quality of care from a patient perspective. The hospital is one of the largest employers in the region with approximately 6500 employees and it provides highly specialized care for 640,000 inhabitants and basic hospital functions for 250,000 inhabitants. The reorganization was focusing on the coordination of patient flows through the hospital. It was initiated by the board of the regional health administration in cooperation with the management board of the university hospital. An important part of this reorganization process was to introduce a new management structure at the department level of the university hospital and to develop a team structure at the ward, specialty and patient levels of each department. Another important part of the reorganization was to develop the links between the hospital and the primary sector, including the general practitioners and the social services of the local communities. Thus, the reorganization was aiming to improve patient flows not only within the university hospital but also between the hospital and the primary sector.

The reorganization of the university hospital has been followed during 2013 and 2014 by a group of organizational researchers (the authors). The members of the group have made a number of interviews with professionals and managers from different clinical departments and different organizational levels within the hospital and also from the management board of the regional health administration. There were five interviews with the top management of the hospital and the regional administration and eight interviews with key persons involved in the reorganization process. In addition, there were two focus group interviews with physicians, nurses and managers from different clinical departments within the hospital.

All the interviews were semi-structured, focusing mainly on the interprofessional and interorganizational integration in the new organization. The data from the interviews were processed in accordance with qualitative methodology, which means that the interviews were transcribed, interpreted and analyzed with respect to their contents [41]. The interpretation and analysis of the data were made in the terms of the organizational models of the conceptual framework in order to illustrate these models. The interview data were also combined with data from observations of meetings and studies of documents like organizational diagrams, statistics and annual reports [42]. These data were also interpreted and analyzed in terms of the different organizational models of the conceptual framework. The following account of the reorganization is based on all of these data.

### Results

The new organizational structure consists of eight clinical departments, each of them with a combination of medical specialties conducive to the main patient flows within the university hospital. This means that the clinical structure is crossing the boundaries of different medical specialties. The main idea behind this structure is to put the patients in the centre of the organization by grouping specialists around the patients instead of sending the patients around to different specialists. Each clinical department is managed by a leadership troika consisting of a clinical director and two clinical vice directors; one of them is responsible for the human resources while the other is responsible for the patient flows of the department. Five clinical directors are physicians, two are administrators and one is a nurse. All the vice directors responsible for human resources are nurses, while there are three physicians and six nurses who are vice directors responsible for patient flows. One of the clinical departments has two vice directors for the patient flows because of the size of the department.

**Figure 1.**
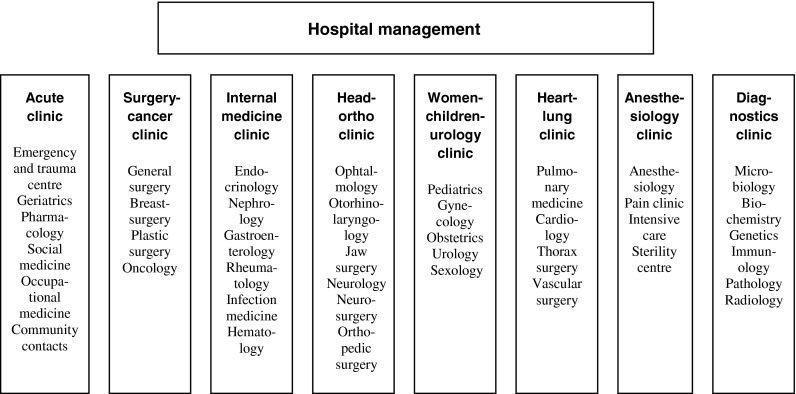
The new clinical structure of the university hospital.

There are three main innovations in this organization. The first innovation is a clinical structure with a creative combination of different medical specialties in order to facilitate the patient flows of the university hospital. Among these can be mentioned ‘the head-ortho clinic’ with a combination of orthopaedics and different specialties of the head including neurology and neurosurgery, ‘the women-children and urology clinic’ with a combination of paediatrics, gynaecology, obstetrics and urological surgery and ‘the heart-lung clinic’ with a combination of pulmonary medicine, cardiology and thorax surgery. One of the clinical departments, ‘the acute clinic’, has an unusual combination of emergency care and geriatrics, but it is also responsible for the contacts between the hospital and the different organizations of the primary sector. The new clinical structure of the hospital is illustrated in Figure 1.

The second innovation is the appointment of clinical vice directors responsible for patient flows. These so-called flow managers are responsible for coordinating the patient flows within their clinical departments and they are sharing the responsibility for coordinating patient flows across the boundaries of the different departments. They are also responsible for coordinating patient flows across the boundaries of the hospital and the primary sector. In this connection, the flow manager of ‘the acute clinic’ has a special task to facilitate the contacts with the organizations of the primary sector. There has been a lively discussion within the hospital on whether the flow managers should be physicians or nurses. All the positions were advertised, but there were few physicians who applied. However, when the flow managers were going to be appointed, many physicians suddenly realized that they would be in a subordinate position to the nurses who had applied for the position. This has led to some opposition from the physicians and there is still a discussion going on concerning the role and the power of the flow managers in the new organization.

**Figure 2.**
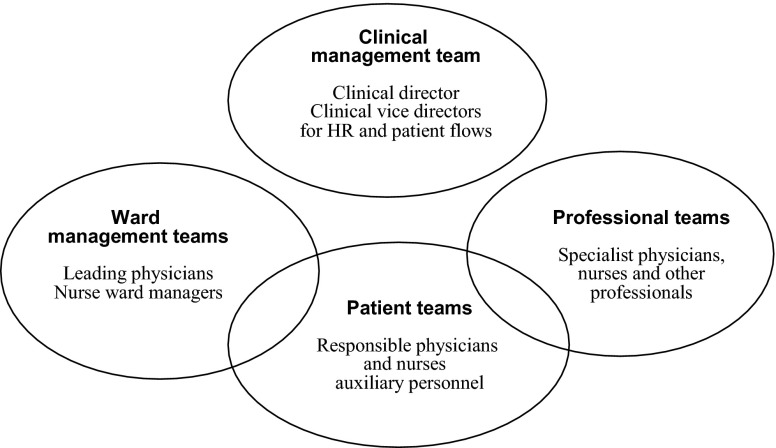
The team structure at the department level of the hospital.

The third innovation is the strong focus on teamwork to support the patient flows of the hospital. The leadership troika at the department level is working as a management team, where the vice director responsible for patient flows has an important role. Within the clinical departments, there is also an ongoing development of interlocking teams on different levels. There are ‘ward management teams’, consisting of senior physicians and nurses in the role of ward managers, who are responsible for the wards and surgeries of the clinical departments. There are also ‘professional teams’ of specialist physicians, nurses and other professionals who are working within or between the different medical specialties of the departments. At the same time, members of these teams are participating as patient responsible physicians and nurses together with auxiliary personnel in ‘patient teams’, which are formed for the operational coordination of care around individual patients. It is in these teams that the improvement of the patient flows is taking place. The team structure at the department level of the university hospital is illustrated in Figure 2.

In addition to these organizational innovations, a number of networks have been established to support the management structure of the new hospital organization. There are, for example, networks for the clinical directors and for the different vice directors. The network of the flow managers is meeting regularly to exchange information and discuss common problems in the coordination of the patient flows. There are also regular meetings between this network and members of the management board of the hospital for evaluation and planning of different activities to facilitate and improve the patient flows. There are still traditional systems of accounting and budgeting in the university hospital, which means that resources are allocated to organizational units instead of activities or processes, but the top management of the hospital is well aware of the need to change these systems in order to implement the new organization and improve the patient flows of the hospital.

## Discussion

The reorganization of the Danish university hospital has not been following the management fashion of many other hospitals to import industrial models of quality management focusing on measurement of time and costs. Instead the university hospital has been more concerned with the organization and management of patient care across professional and organizational boundaries. The new organization of the hospital contains many elements from the different models of integrated care. The whole organization is based on a matrix way of thinking, combining the horizontal integration of patient flows with the vertical integration of clinical departments. This is even more the case with patient flows across different departments or across the great divide between the hospital and the primary sector. At the same time, the combination of specialties within the departments can be regarded as a form of horizontal integration across the vertical integration of the traditional medical disciplines.

According to the literature of organization and management, a matrix organization can be an efficient way to integrate new activities or organizational units within a hierarchical structure, but it is also a complicated and fragile structure that may lead to confusion, stress and conflicts about leadership. This is particularly the case in a professional organization, like a hospital, where there are often boundary conflicts between different professions involved [43, 44]. Therefore, the matrix structure needs to be supported by different ‘liaison devices’ like task forces, standing committees or integrating managers [25]. Many of these devices are included in the different organizational models of integration.

There are elements of interprofessional as well as interorganizational integration in the new organization of the university hospital. There is a strong focus on teamwork on different levels of the organization. There are management teams on the department level and the ward level, which consist of managers with different professional backgrounds. In the clinical departments, there are multidisciplinary teams of professionals working within the different medical specialties of the departments or across these specialties. In addition, the members of these teams are also members of other multidisciplinary teams, which are formed around individual patients or groups of patients. This means a complicated structure with overlapping or interlocking multidisciplinary teams, which contributes to the interprofessional as well as the interorganizational integration of the hospital [31].

Despite the risk of professional boundary conflicts, the different management and multidisciplinary teams provide ample opportunities for consultations and information exchange between different managers and different professionals in the new hospital organization. In addition, a number of networks have been established for key management functions in the new organization. There are networks for the clinical directors and the different vice directors. Within these networks, there are regular meetings to exchange information and discuss common problems. There are also regular meetings between these networks and the hospital management. All of these meetings are important devices for the integration of the organization [27].

The appointment of clinical vice directors responsible for patient flows is another integration device. These flow managers have an important role as integrating managers in the matrix organization of the hospital [26]. They can be regarded as a sort of case managers for different patient groups. The flow managers are responsible for the coordination of patient flows within their respective clinical departments and between different departments of the hospital as well as between the hospital and the primary sector. They have a strategic responsibility for these patient flows, while the patient teams are responsible for the operational coordination of care around individual patients. It is a difficult task to coordinate the work of professionals, particularly when they are physicians with a legal responsibility for their patients. This task is made even more difficult by the resistance of the physicians to be managed by nurses as flow managers. Therefore, the coordination of patient flows involves negotiations with physicians as well as elimination of practical or administrative barriers and obstacles [45].

There are no formal agreements on collaboration between the different clinical departments in the hospital organization, but such agreements may be necessary if the departments become stronger and more independent as organizational units. In the same way, it may be necessary to develop existing agreements with the general practitioners and the local communities into formal partnerships in order to deal with patient flows that are stretching out to the primary health care and the social services of the local communities [34].

There has been a development towards a co-location of different medical specialties within the clinical departments, but this development has not gone so far because of the limitations of the existing hospital buildings. The only department where all the specialties are co-located is ‘the internal medicine clinic’, which is placed in a separate hospital building. In a few years, however, the university hospital will be moving to new buildings and these buildings are being planned with the new hospital organization in mind.

The most far-reaching model of interorganizational integration is financial coordination. This means that resources from different organizations or organizational units are pooled in order to finance common activities or processes [36, 37]. Such coordination may eliminate many barriers to collaboration and integration related to territoriality and competition for resources [46]. In the new hospital organization, this means that resources from the clinical departments could be coordinated to finance the patient flows across the boundaries of the different departments. Since finance is a powerful incentive, it would contribute to strengthen the coordination of the patient flows. The hospital management is aware of the need to change the systems of accounting and budgeting in the hospital, but they have first concentrated on implementing the new organization structure. Maybe it would have been a better strategy to start with the financial control systems instead of trying to change them afterwards.

## Concluding remarks

The description and discussion of the reorganization of the university hospital have shown that integrated care can be a relevant concept not only for health promotion and primary health care, where the concept was first developed, but also for a highly specialized hospital organization. The new organization of the Danish university hospital has been analyzed in terms of different organizational models of integrated care. From the analysis, it is clear that there are many elements of interprofessional integration in the new organization, for example, the strong focus on multidisciplinary teamwork and meetings, providing many opportunities for consultation and information exchange between professionals on different levels of the organization. There are also some important elements of interorganizational integration, for example, the appointment of flow managers as a sort of case managers and the intentions to co-locate different specialties within the clinical departments.

The new organization was designed to improve the patient flows of the university hospital by grouping specialists around the patients instead of sending the patients around to different specialists. This means that the organization is oriented towards processes of care instead of the traditional orientation towards structures like clinical departments or medical specialties. Such a reorientation may challenge established professional identities and boundaries. The opposition from the physicians against the appointment of nurses as flow managers can be understood as a reaction against this reorientation. However, it can also be understood as a power struggle between two professional groups in the hospital. In any case, the opposition is hampering the implementation of the new organization. In addition, the implementation is made more difficult by the existing systems of accounting and budgeting, where the resources are allocated to the clinical departments rather than the patient flows. This means that the financial incentives are working against the coordination of the patient flows.

Resistance to change is not surprising in a professional organization like a university hospital. The implementation of the new organization may therefore be an uphill battle before the role of the flow managers has been accepted and the financial control systems have become more process oriented. These are important questions for further research and development. It seems, however, that the design of the new organization is basically sound and in line with the organizational models of integrated care, which may contribute to improve the patient flows of the university hospital. As mentioned before, it is not clear what the fashionable industrial models of quality management have actually achieved in health care, so maybe the concept of integrated care is a more promising way to improve the quality of care than the industrial models of quality management. At the same time, the application of this concept in a university hospital may also contribute to widen the traditional field of integrated care.
